# Supplementary material for: Spatiotemporal analysis of SARS-CoV-2 infection reveals an expansive wave of monocyte-derived macrophages associated with vascular damage and virus clearance in hamster lungs
Source: Microbiol Spectr. 2023 Nov 27;12(1):e02469-23. doi: 10.1128/spectrum.02469-23 (PMC10782978; doi:10.1128/spectrum.02469-23)
Supplement: Tables S1 to S3 — Animal observation scoring; Histological scoring criteria; Primary antibodies used for IHC, applications, special staining and image analysis. [file spectrum.02469-23-s0001.docx]

Table S1. Animal observation scoring. For each criterion, a maximum of 3 points was possible per animal (i.e. 18 animals: maximum score of 54).

| dpi | animals | activity | posture | respiration | movement | condition | food intake | fur | eyes | feces/urine | Total clinical signs |
| --- | --- | --- | --- | --- | --- | --- | --- | --- | --- | --- | --- |
| 0 | 24 | 0 | 0 | 0 | 0 | 0 | 0 | 0 | 0 | 0 | 0 |
| 1 | 24 | 0 | 0 | 0 | 0 | 0 | 0 | 0 | 0 | 0 | 0 |
| 2 | 21 | 0 | 0 | 0 | 0 | 0 | 0 | 0 | 0 | 0 | 0 |
| 3 | 18 | 3 | 0 | 1 | 0 | 0 | 0 | 0 | 0 | 0 | 4 / 486 |
| 4 | 15 | 11 | 0 | 7 | 0 | 0 | 0 | 5 | 0 | 0 | 23 / 405 |
| 5 | 12 | 9 | 0 | 2 | 0 | 0 | 0 | 9 | 0 | 0 | 20 / 324 |
| 6 | 9 | 6 | 0 | 6 | 0 | 0 | 0 | 6 | 0 | 0 | 18 / 243 |
| 7 | 6 | 0 | 0 | 3 | 0 | 0 | 0 | 1 | 0 | 0 | 4 / 162 |

Table S2. Histological scoring criteria

| **No.** | **Criteria** | **Scoring description*** |
| --- | --- | --- |
| 1 | activation blood vessel (hypertrophy, rolling of immune cells, degeneration, subendothelial immune cells) | present =1, dominant 3= >3 in 5 mm^2^ lung tissue |
| 2 | vasculitis (necrosis, intramural immune cells) |  |
| 3 | vascular occlusion by thrombus or disruption |  |
| 4 | edema, perivascular | affected area / lung, 0= no lesion; 1= <5%; 2= 6%–40%; 3 = 41%–80%; 4 =>80% |
| 5 | edema, alveolar |  |
| 6 | edema, peribronchial |  |
| 7 | edema, interstitial |  |
| 8 | hemorrhage, alveolar |  |
| 9 | hemosiderin | present = 1 |
| **Sum 1-9** | **vascular lesion score** |  |
| 10 | necrotizing bronchitis, area | affected area / lung, 0= no lesion; 1= <5%; 2= 6%–40%; 3 = 41%–80%; 4 =>80% |
| 11 | necrotizing bronchitis, grade | 1 = single cells, 2 = aggregates of cells, 3 = almost filling the lumen; 4 = luminal filled^a^ |
|  | predominant cell type | h-heterophil, l-lymphocyte, m-macrophage, p-plasma cell |
| 12 | inflammation bronchointerstitial, area | affected area / lung, 0= no lesion; 1= <5%; 2= 6%–40%; 3 = 41%–80%; 4 =>80% |
| 13 | inflammation bronchointerstitial, grade | 1 = 1 cell layer, 2 = 2-3, 3 = 4-5, 4 ≥ 6 layers |
|  | predominant cell type | h-heterophil, l-lymphocyte, m-macrophage, p-plasma cell |
| 14 | infiltrates perivascular, area | affected area / lung, 0= no lesion; 1= <5%; 2= 6%–40%; 3 = 41%–80%; 4 =>80% |
| 15 | infiltrates perivascular, grade | 1 = 1 cell layer, 2 = 2-3, 3 = 4-5, 4≥6 layers |
|  | predominant cell type | h-heterophil, l-lymphocyte, m-macrophage, p-plasma cell |
| 16 | inflammation interstitial, area | affected area / lung, 0= no lesion; 1= <5%; 2= 6%–40%; 3 = 41%–80%; 4 =>80% |
| 17 | inflammation interstitial, grade | 1 = 1 cell layer, 2 = 2-3, 3 = 4-5, 4≥6 layers |
|  | predominant cell type | h-heterophil, l-lymphocyte, m-macrophage, p-plasma cell |
| 18 | infiltrates alveolar, area | affected area / lung, 0= no lesion; 1= <5%; 2= 6%–40%; 3 = 41%–80%; 4 =>80% |
| 19 | infiltrates alveolar, grade | 1 = single cells, 2 = aggregates of cells, 3 = almost filling the lumen; 4 = luminal filled |
|  | predominant cell type | h-heterophil, l-lymphocyte, m-macrophage, p-plasma cell |
| 20 | atelektasis, inflammation associated, area | affected area / lung, 0= no lesion; 1= <5%; 2= 6%–40%; 3 = 41%–80%; 4 =>80% |
| **Sum 10-20** | **inflammation score** |  |
| 21 | necrosis, secondary bronchi, area | affected area / lung, 0= no lesion; 1= <5%; 2= 6%–40%; 3 = 41%–80%; 4 =>80% |
| 22 | necrosis, secondary bronchi, grade | affected area / brochus, 0= no lesion; 1= <5%; 2= 6%–40%; 3 = 41%–80%; 4 =>80% |
| 23 | necrosis alveolar epithelium | affected area / lung, 0= no lesion; 1= <5%; 2= 6%–40%; 3 = 41%–80%; 4 =>80 affected area / lung |
| 24 | diffuse alveolar damage (AEC necrosis, hyaline membrane, debris, fibrin) |  |
| **Sum 21-24** | **necrosis score** |  |
| 25 | hypertrophy/hyperplasia, bronchi with basophilia, multinucleated cells | affected area / lung, 0= no lesion; 1= <5%; 2= 6%–40%; 3 = 41%–80%; 4 =>80 |
| 26 | hyperplasia bronchiolo-alveolar (bronchiolization) |  |
| 27 | atypical cells (multinucleated), alveolar | present = 1 |
| 28 | fibrosis, interstitial, diffuse | present = 1 |
| **Sum 25-28** | **regeneration score (criteria 25-28)** |  |
| **SUM 1-28** | **TOTAL LESION SCORE** |  |

AEC: alveolar epithelial cell

^a^predominant grade on slide selected

*scores can be translated to minimal (score 1), mild (score 2), moderate (score 3), severe (score 4)

Table S3. Primary antibodies used for IHC, applications, special staining and image analysis including measurement parameters

| **Immunohistochemistry** | | | | |
| --- | --- | --- | --- | --- |
| **Marker** | **Antibody** | **Pre-treatment** | **Secondary reagents** | **Measurement parameter** |
| SARS-CoV NP | Rabbit anti- SARS-CoV NP(Novus Biologicals,# NB100-56576), 1:200, ON | HIER, Citrate buffer pH 6.0, for 20 min | Anti-rabbit IgG Biotinylated (Vector Laboratories), 1:200, 30 min. RT; and ABC Kit Vectastain Elite PK 6100, 30 min, RT (Vector Laboratories) | Positive area per total tissue area |
| AIF1 | Rabbit anti-Iba1 (FUJIFILM Wako Pure Chemical Corporation, #019-19741), 1:800, ON | HIER, Citrate buffer pH 6.0, for 20 min | Dako EnVision+ System- HRP Labelled Polymer Anti-rabbit, 30 min, RT | Positive area per total tissue area |
| TTF-1 | Rabbit anti-TTF1  (abcam, ab76013), 1:100, ON | HIER, Citrate buffer pH 6.0, for 20 min | Anti-rabbit IgG Biotinylated, 1:200, 30 min. RT; and ABC Kit, 30 min, RT | Positive cells per total tissue area |
| **Special staining** | | | | |
| **Name** | **Kit / Chemicals** |  |  | **Measurement parameter** |
| Azan stain | Heidenhain's AZAN trichrome stain kit, following user manual (MORPHISTO, #12079 00250) | | | Positive area per total tissue area |
| Prussian blue | Hydrochloric Acid Solution, Potassium ferrocyanide solution, Nuclear fast red-aluminum sulfate solution | | | 1 = 1-3 foci  2= >3 foci  3 = confluent  4 diffuse |

Abbreviations: IHC, immunohistochemistry; PAX, paired box protein; CD, cluster of differentiation; AIF1, ionized calcium-binding adapter molecule 1; HIER, Heat induced epitope retrieval; EDTA, Ethylenediamine tetraacetic acid; RT, room temperature; ON, overnight at 4°C
