# Supplementary material for: Spatiotemporal analysis of SARS-CoV-2 infection reveals an expansive wave of monocyte-derived macrophages associated with vascular damage and virus clearance in hamster lungs
Source: Microbiol Spectr. 2023 Nov 27;12(1):e02469-23. doi: 10.1128/spectrum.02469-23 (PMC10782978; doi:10.1128/spectrum.02469-23)
Supplement: Fig. S1 to S8 — Supplementary figures and legends. [file spectrum.02469-23-s0002.pdf]

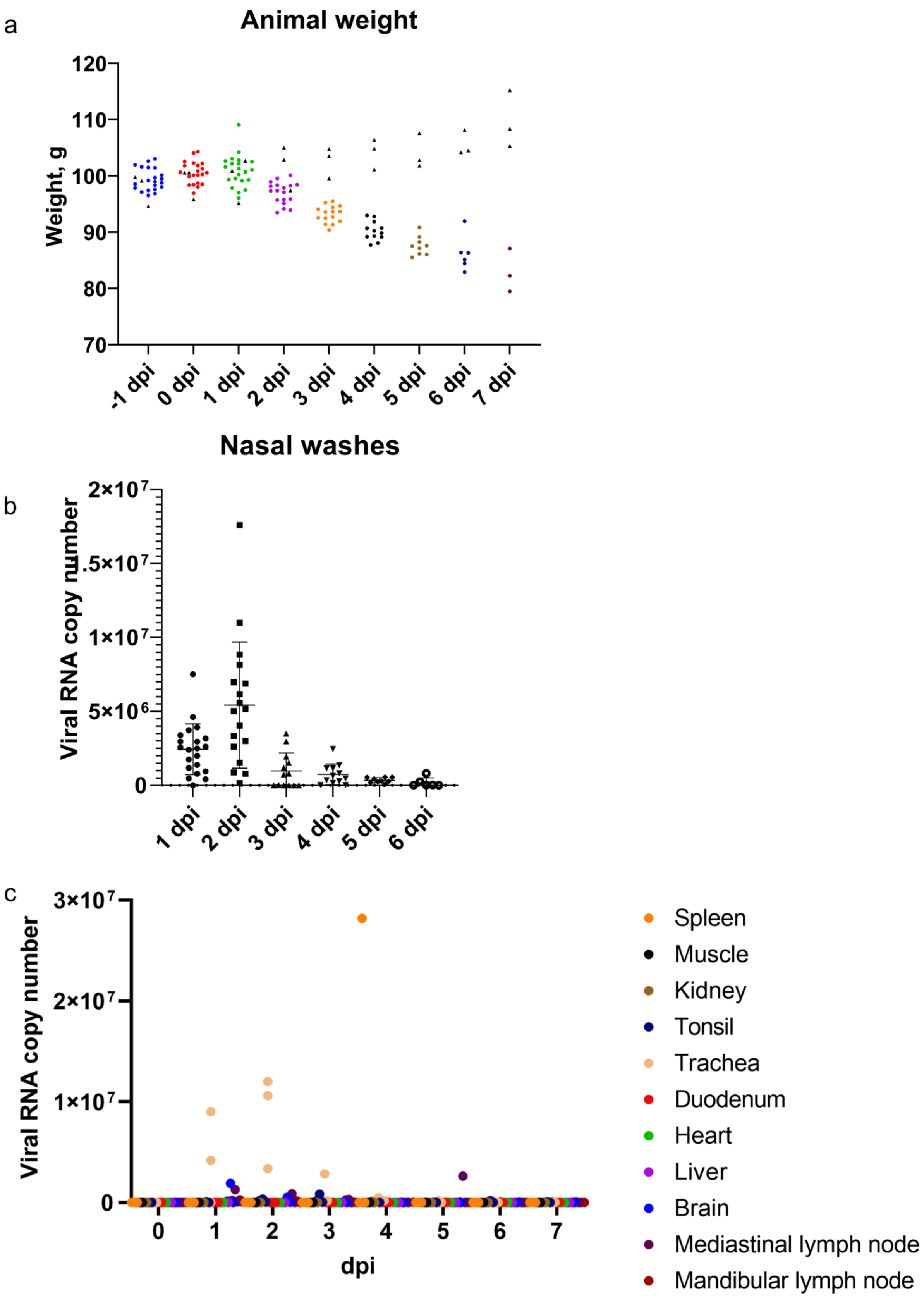
a
Animal weight
Weight, g
120
110
100
90
80
70
-1 dpi
0 dpi
1 dpi
2 dpi
3 dpi
4 dpi
5 dpi
6 dpi
7 dpi
Nasal washes
b
Viral RNA copy number
2×10^7
1.5×10^7
1×10^7
5×10^6
0
1 dpi
2 dpi
3 dpi
4 dpi
5 dpi
6 dpi
c
Viral RNA copy number
3×10^7
2×10^7
1×10^7
0
0
1
2
3
4
5
6
7
dpi
Spleen
Muscle
Kidney
Tonsil
Trachea
Duodenum
Heart
Liver
Brain
Mediastinal lymph node
Mandibular lymph node

**Figure S1. Animal weight and PCR analysis of viral RNA in nasal washes and different tissues.** a) Animal weight measurements during the infection time-course. All animals were weighed one day prior infection (-1 dpi), on the day of infection (0 dpi) and on the following days. Control animal data is shown in black triangles. b) PCR analysis of viral RNA copy number ($Log_{10}$) in nasal washes. c) PCR analysis of viral RNA copy number (Log10) in examined tissues. Each point in b and c represents data from a nasal wash or approximately 1.5-2 $mm^3$ tissue sample, or the entire tissue in case of lymph nodes, of one animal.

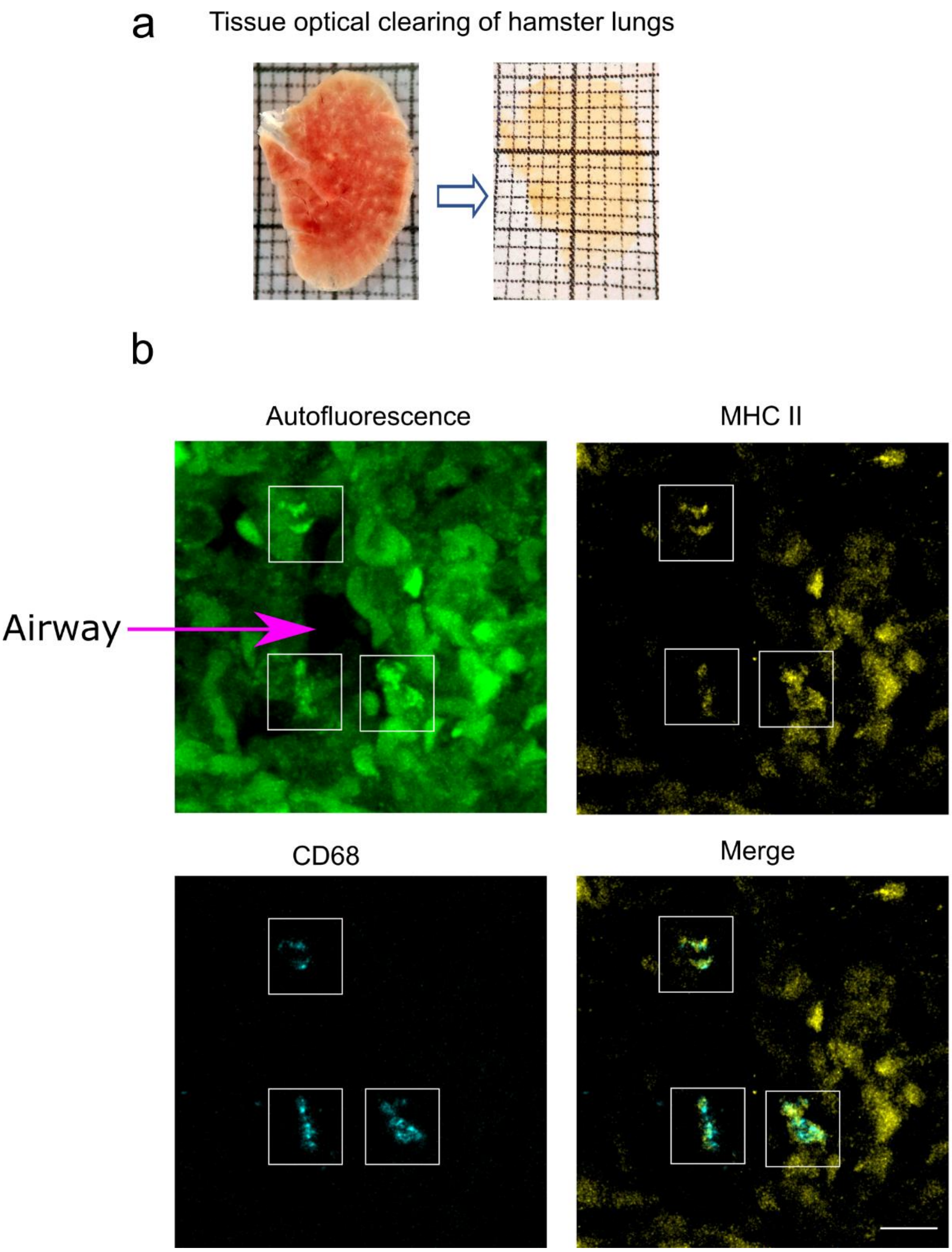


**Figure S2. Tissue optical clearing and alveolar macrophage characterization in control lung tissues.** a) Representative images of hamster lung tissue before (left) and after clearing (right) are shown b) Confocal imaging of control lungs showing alveolar macrophages. Maximum intensity projections of autofluorescence at 488 nm (green), CD68 (cyan), MHC II (yellow), and a merge of MHC II and CD68. White rectangles indicate location of $CD68^{+}$, MHC $II^{+}$ alveolar macrophages. Scale bar 10 μm.

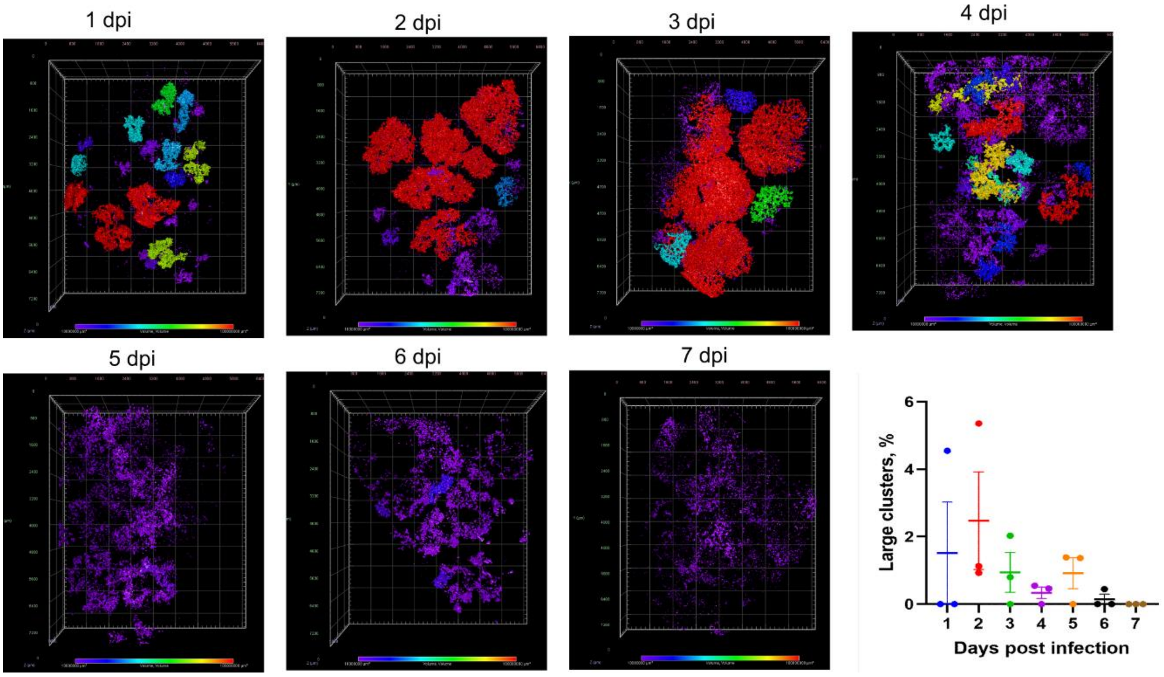


**Figure S3. SARS-CoV-2 node size distribution during the infection time-course.** a) The NP fluorescence of samples from 1 to 7 dpi shown in Fig. 2a was classified according to the NP cluster size and displayed as 3D color-coded heatmap. b) Distribution of large NP clusters shown as a fraction of total cluster number from 1 to 7 dpi.

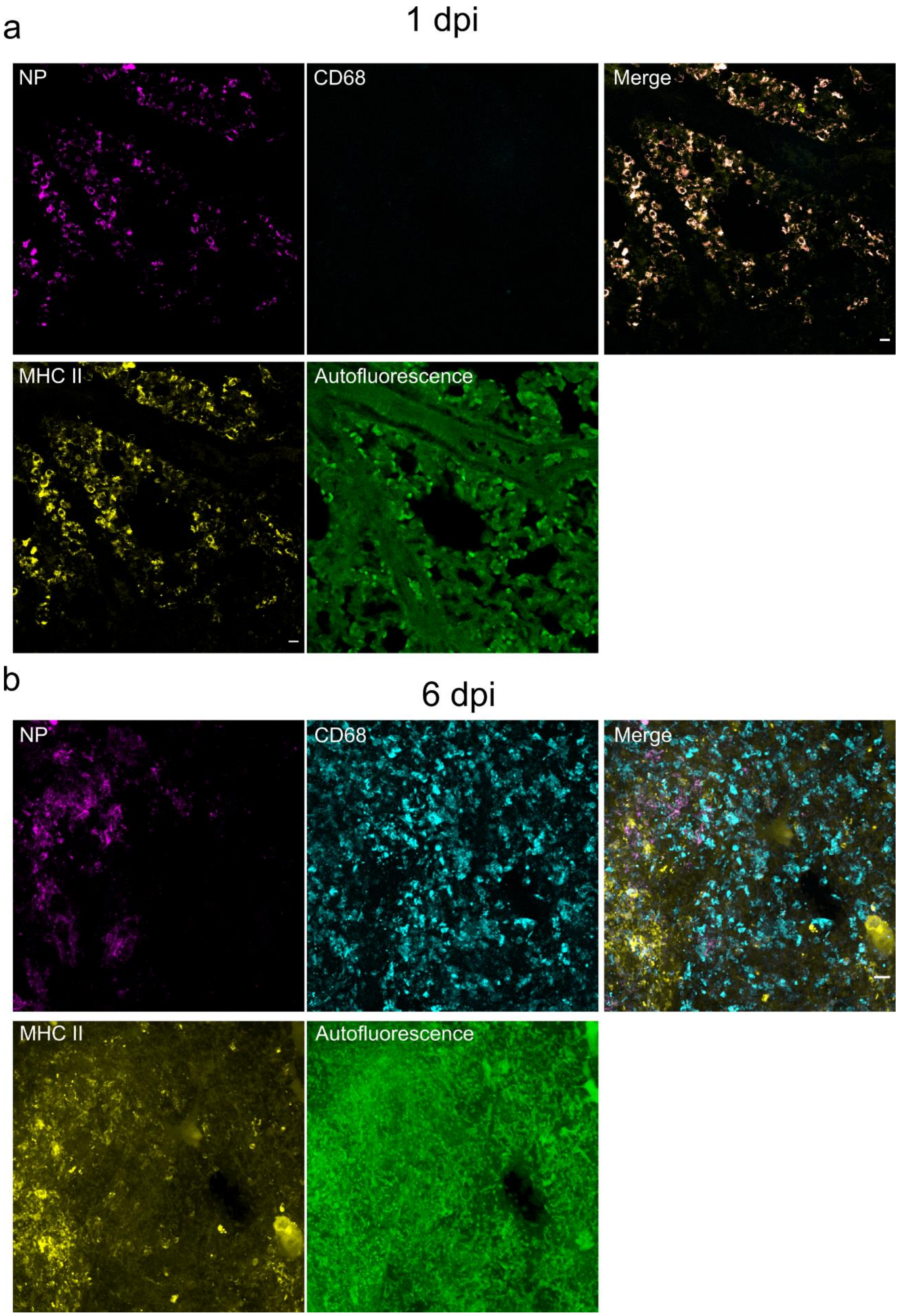


**Figure S4. Confocal imaging of lung infection, MHC II and CD68 at 1 dpi (a) and 6 dpi (b).** Maximum intensity projections of NP (magenta), CD68 (cyan), MHC II (yellow), autofluorescence at 488 nm (green). Scale bar 50 μm.

a

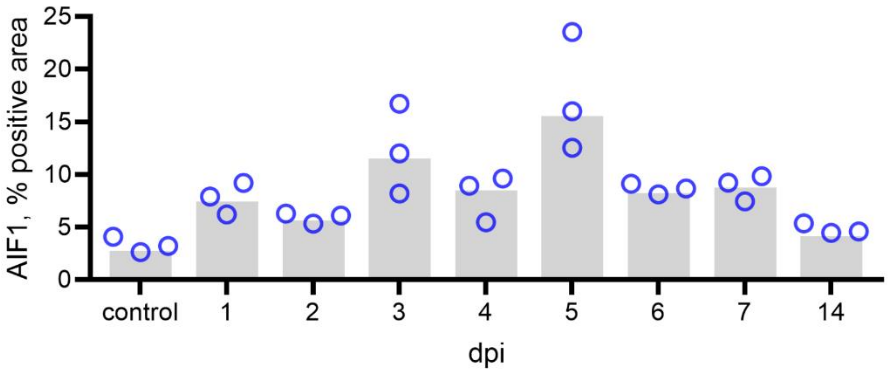

AIF1, % positive area
25
20
15
10
5
0
control
1
2
3
4
5
6
7
14
dpi


b

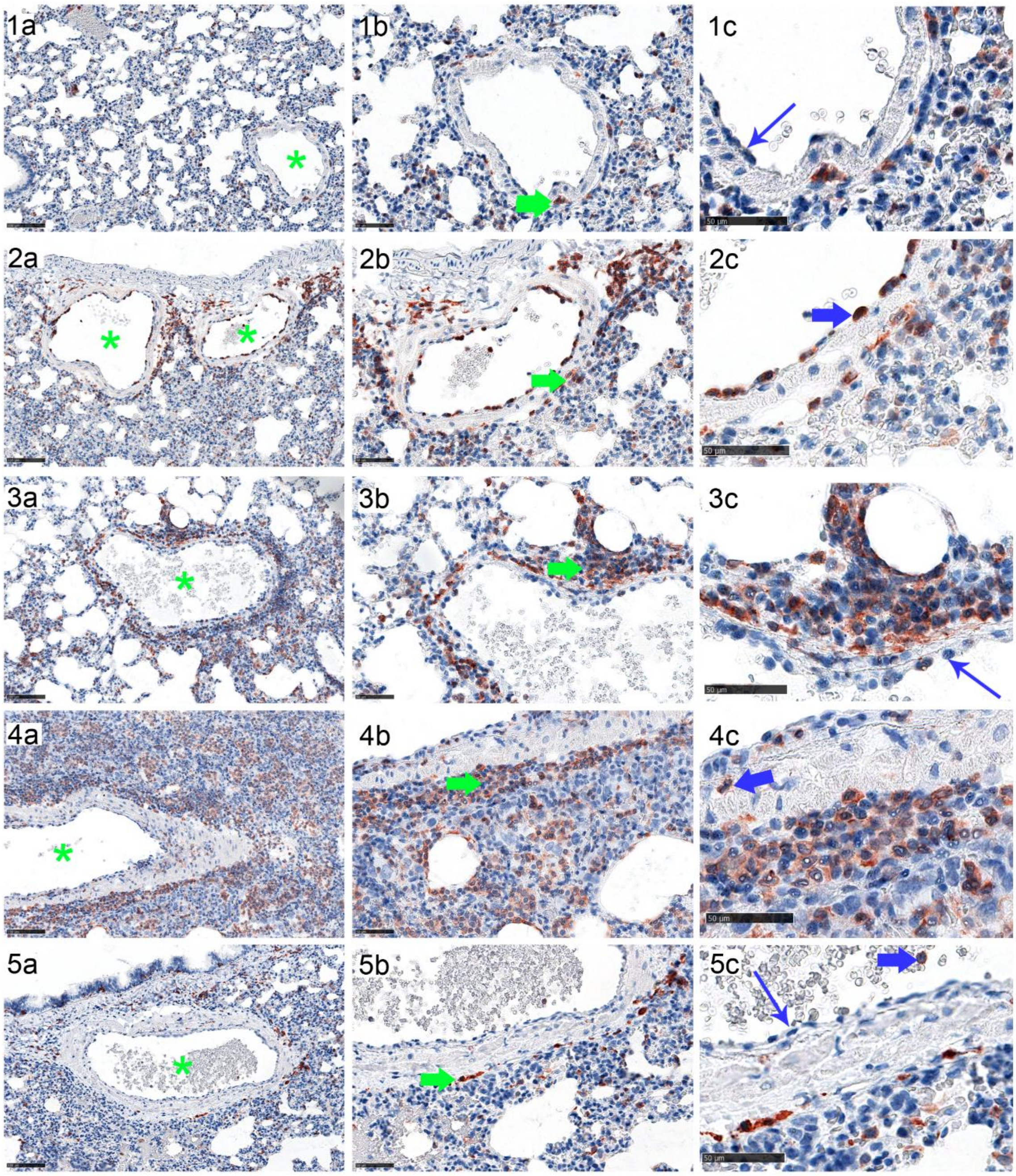

1a
1b
1c
2a
2b
2c
3a
3b
3c
4a
4b
4c
5a
5b
5c

**Figure S5. Spatiotemporal distribution of macrophages. (a) Immunohistochemistry (IHC) and quantitative 2D image analysis on AIF1 labelled lung slides for temporal evaluation.** Waves of macrophage influx peaking on day 5 and almost reaching the baseline on day 14. HALO software (Version: 3.2.1851.439, Indica Labs), Area Quantification v2.1.11 module. Dots, relative AIF-positive area results for individual animals; bar, group median. **(b) Representative pictures showing IHC for AIF1 for spatial evaluation.** In control animals (**1**), Day 1 (**2**), day 5 (**3** less affected, **4** strongly affected area) and day 14 (**5**) post infection. Pictures showing an overview with perivascular (**a**, green asterisk indicating blood vessel lumen) and intra-alveolar macrophages (**a**, blue asterisk) with cytoplasmic AIF1 labelling. Image detail indicating perivascular macrophages (**b**, green arrow), image detail showing unlabeled endothelial cells (**1c, 3c, 5c**, blue slim arrow), and AIF1-positive monocytes/macrophages either intravascularly (**2c**, **5c** blue thick arrow) or transmigrating (**4c** blue thick arrow). Note that one day post infection, monocytes/macrophage constitute the main immune cell population involved in immune cell “rolling” (2c). Immunohistochemistry, AIF1 antigen, ABC Method, AEC chromogen (red), hematoxylin counterstain (blue), bar 100 µm (a) and 50 µm (b-c).

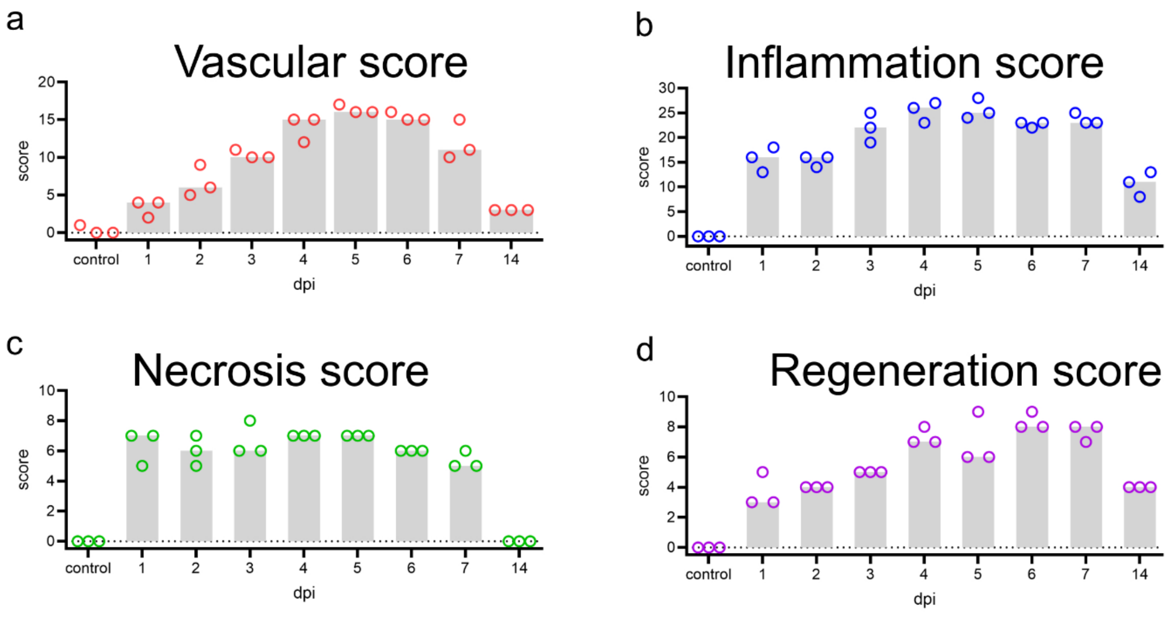


**Figure S6. Detailed lung lesion scores.** Inflammatory (a), vascular (b), necrosis (c) and regeneration (d) scoring was performed using scoring criteria described in Table S2.

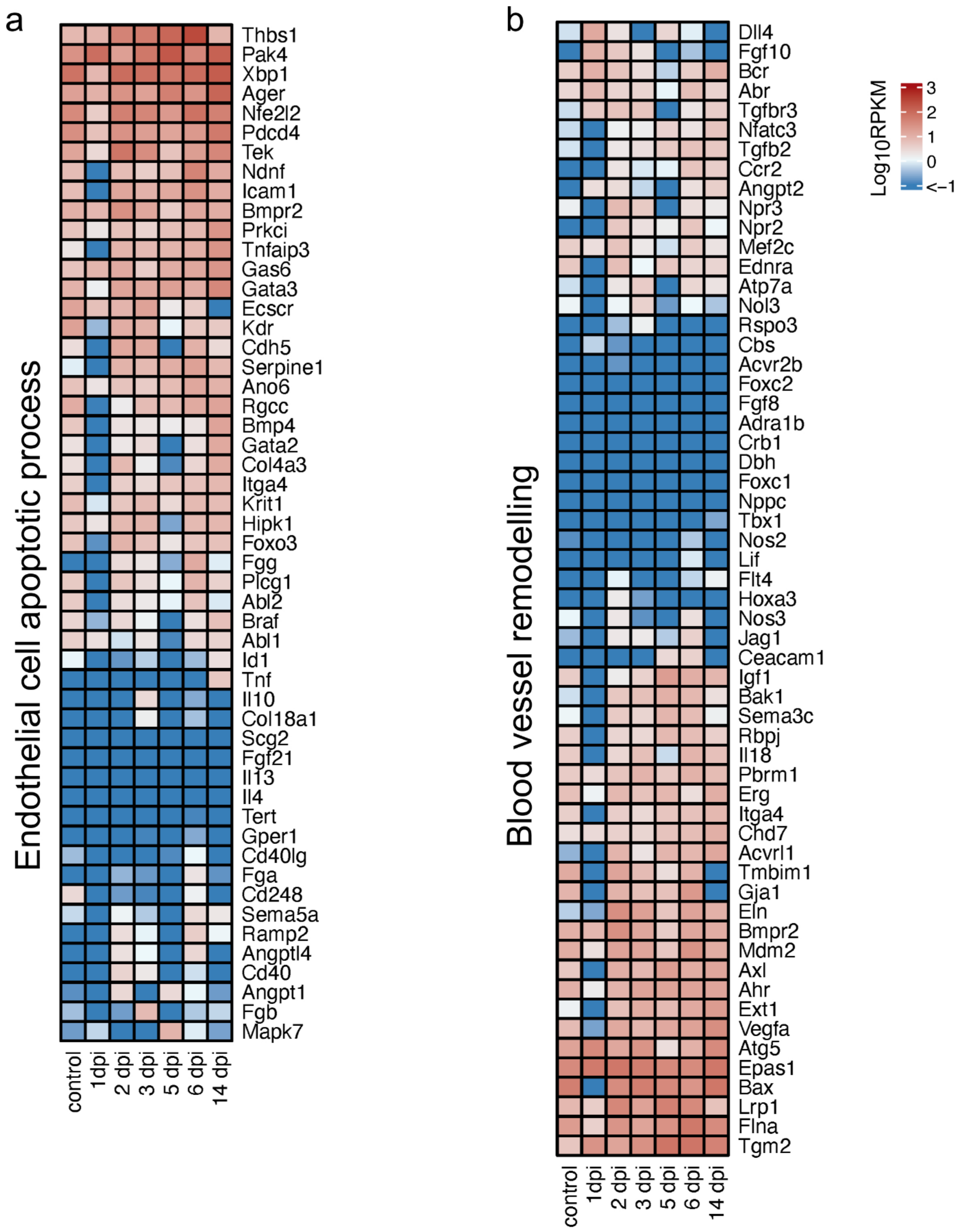


**Figure S7. Heat maps depicting scaled mRNA expression of endothelial apoptosis and vessel remodeling.**

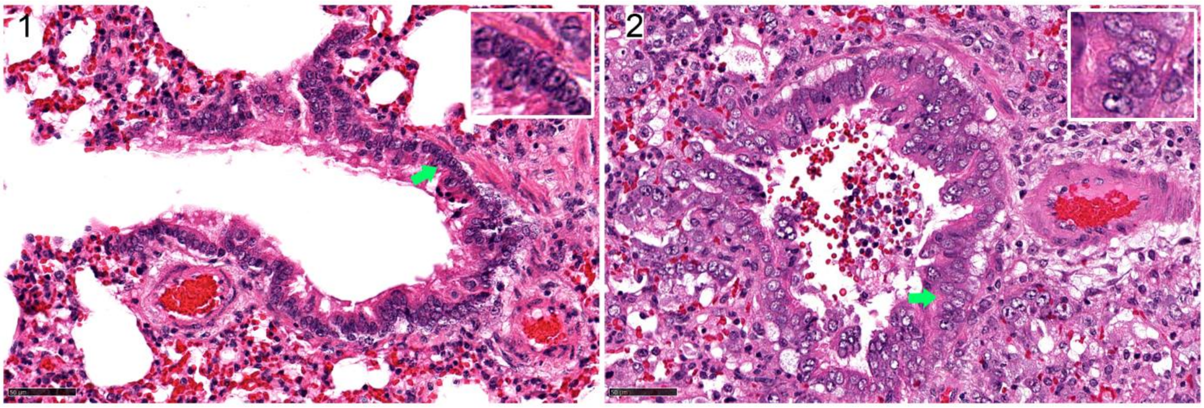


**Figure S8. Regeneration of conducting airways.** Control animals present with pseudostratified columnar epithelium, containing round to oval heterochromatic (inlay) nuclei in terminal airways (**1**). SARS-CoV-2 infection associated damage of the bronchial epithelium leads to regenerative hyperplasia and hypertrophy starting on day 2, here exemplarily shown on day 6 (**2**). Cells exhibit euchromatic (inlay) nuclei. Hematoxylin eosin stain, bar 50 µm.
